# Genome-wide identification of quantitative trait loci in a cross between Hampshire and Landrace II: Meat quality traits

**DOI:** 10.1186/1471-2156-9-22

**Published:** 2008-02-28

**Authors:** Ellen Markljung, Martin H Braunschweig, Peter Karlskov-Mortensen, Camilla S Bruun, Milena Sawera, In-Cheol Cho, Ingela Hedebro-Velander, Åsa Josell, Kerstin Lundström, Gertrud von Seth, Claus B Jørgensen, Merete Fredholm, Leif Andersson

**Affiliations:** 1 Department of Medical Biochemistry and Microbiology, Uppsala University, Box 597, SE-751 24 Uppsala, Sweden; 2 Department of Animal Breeding and Genetics, Swedish University of Agricultural Sciences, Box 597, SE-751 24 Uppsala, Sweden; 3 Institute of Genetics, Vetsuisse Faculty, University of Berne, Switzerland; 4 Department of Animal and Veterinary Basic Sciences, Division of Genetics and Bioinformatics, Faculty of Life Sciences, University of Copenhagen, Frederiksberg, Denmark; 5 Department of Animal Science, Warsaw Agricultural University, Ciszewskiego 8, 02-786 Warsaw, Poland; 6 National Institute of Subtropical Agriculture, R.D.A., 175-6, O-deung dong, Jeju, 690-150, South Korea; 7 Quality Genetics, 244 82 Kävlinge, Sweden; 8 Ugglarps slakteri AB, PI 91, SE-231 96, Trelleborg, Sweden; 9 Department of Food Science, Swedish University of Agricultural Sciences, Box 7051, SE-750 07 Uppsala, Sweden; 10 Tetra Pak Research & Development AB, Ruben Rausings gata, SE-221 86 Lund, Sweden

## Abstract

**Background:**

Meat quality traits are important in pig breeding programs, but they are difficult to include in a traditional selection program. Marker assisted selection (MAS) of meat quality traits is therefore of interest in breeding programs and a Quantitative Trait Locus (QTL) analysis is the key to identifying markers that can be used in MAS. In this study, Landrace and Hampshire intercross and backcross families were used to investigate meat quality traits. Hampshire pigs are commonly used as the sire line in commercial pig breeding. This is the first time a pedigree including Hampshire pigs has been used for a QTL analysis of meat quality traits.

**Results:**

In total, we analyzed 39 meat quality traits and identified eight genome-wide significant QTL peaks in four regions: one on chromosome 3, two on chromosome 6 and one on chromosome 16. At least two of the QTLs do not appear to have been detected in previous studies. On chromosome 6 we identified QTLs for water content in *M. longissimus dorsi *(LD), drip loss in LD and *post mortem *pH decline in LD. On chromosomes 3 and 16 we identified previously undetected QTLs for protein content in LD and for freezing and cooking loss respectively.

**Conclusion:**

We identified at least two new meat quality trait QTLs at the genome-wide significance level. We detected two QTLs on chromosome 6 that possibly coincide with QTLs detected in other studies. We were also able to exclude the C1843T mutation in the ryanodine receptor (*RYR1*) as a causative mutation for one of the chromosome 6 QTLs in this cross.

## Background

Since the first Quantitative Trait Locus (QTL) analysis in pigs was published in 1994 [1], QTL analyses have been widely used to identify chromosomal regions harbouring genes for various complex traits in the pig such as growth, carcass composition and meat quality [2]. Meat quality traits have been studied before using crosses between Wild Boar and Large White [3,4], Meishan and Yorkshire [5], Meishan and Large White/Landrace [6], Duroc and Landrace/Yorkshire [7], Berkshire and Yorkshire [8], Iberian and Landrace [9], Pietrain and Meishan and Wild Boar [10], and between Duroc and Berlin Miniature pig [11].

In this study we used a cross between Finnish Landrace and Swedish Hampshire set up by the Swedish breeding company Quality Genetics, as a combined intercross/backcross design. Landrace has been used in several QTL crosses before, but so far the Hampshire breed has not been used in any QTL intercross which provided an opportunity to detect specific QTL alleles that have been selected in this breed. Landrace and Hampshire pigs differ in a number of traits including coat colour, body composition, fertility and meat quality. Landrace has a long body compared to the shorter more compact Hampshire pig and Hampshire is more muscular than Landrace [12].

A mutation in *PRKAG3*, the RN-mutation (RN^-^), has a large impact on the technological yield and meat quality and has been widespread amongst Hampshire pigs [13]. Its high frequency was most likely the result of its ability to increase the lean meat content of pigs. The effect of the RN-mutation in this cross on traits such as technological yield, meat quality and colour characteristics of pork has been published elsewhere [14-16].

A genome scan detecting QTLs for carcass traits in this cross was published previously [17], and in this study we report the results for meat quality traits. We identified four QTL regions on three different chromosomes that reached genome-wide significance. At least two of these have not been detected in previous studies.

## Results

The total length of the linkage map including all autosomes was estimated to be 23.8 Morgans (M). The average distance between markers was 23.1 cM, with five telomeric regions on SSC 3, 5, 7, 8 and 16 exceeding 50 cM between markers. The linkage map is presented in Table 1.

**Table 1:** Sex average linkage map used for QTL mapping. Distances in Kosambi cM relative to the first marker on the chromosome.

*Chr*.	*Marker*	*Position (cM)*
1	SW1514	0
	SW64	27.1
	S0008	38.6
	SW2035	69.5
	SW962	91.7
	SW1311	113.7
	SW1957	121.3
	SW2512	148.5
2	SW2443	0
	SW1650	29.0
	SW1686	54.0
	SW1517	84.8
	SWR2157	97.9
	SWR345	123.3
	S0036	141.3
3	SW274	0
	SW833	57.4
	SW487	80.4
	SW271	108.5
	SW730	133.9
	S0002	145.6
	SWR2096	173.0
4	S0227	0
	S0301	26.9
	SW2454	51.6
	SW841	68.9
	SW445	97.7
	SWR153	119.6
5	SW491	0
	S0092	56.8
	SW2	71.7
	SW1468	94.8
	SWR1526	111.2
	SW1982	124.0
	SW1954	146.8
	SW967	168.1
6	S0035	0
	SW2535	4.2
	SW1057	41.5
	SW492	69.3
	SW122	92.1
	SW1055	118.4
	S0121	133.6
	SW322	178.4
	SW2419	197.3
7	SW2564	0
	SW1354	20.6
	SW1369	44.4
	SW1409	55.1
	SWR2036	75.7
	SW632	104.0
	S0101	123.8
	SW764	187.5
8	SW2410	0
	SW444	80.0
	S0225	95.4
	SW790	127.5
	S0178	156.9
9	SW983	0
	SW21	12.0
	SW911	32.9
	S0176	55.0
	SW1491	78.8
	SW2093	100.6
	SWR1014	134.3
10	SW830	0
	SW767	28.7
	SW2195	48.6
	S0070	58.5
	SW1991	87.1
	SW951	101.1
	SWR67	122.0
11	S0391	0
	SW2008	17.5
	S0071	47.8
	SW1377	84.7
	SW2413	112.7
12	SW2490	0
	S0229	13.8
	SW957	27.7
	SW168	51.7
	SW62	72.8
	SE259162	98.7
13	S0282	0
	SW935	20.3
	SWR1008	49.8
	SW520	77.3
	SW1056	96.8
	SW2440	105.9
	S0291	126.7
14	SW619	0
	SW510	28.1
	SW2519	49.8
	SW55	81.5
	SW2515	105.8
15	SW2072	0
	SW1562	20.4
	SW1989	44.4
	SW1683	65.0
	PRKAG3	74.8
	SW1983	85.8
	SWR2121	112.3
16	SW2411	0
	SW419	9.1
	SW81	31.3
	SWR2480	45.2
	S0061	101.1
17	PKM	0
	SWR1004	7.8
	SW2441	31.8
	rbdd_67708	39.0
	S0292	53.8
	S0359	65.9
	S0332	89.9
	SW2427	102.7
18	SW1808	0
	SW2540	5.2
	SW1023	15.5
	SW787	34.0
	S0120	51.7
	SY31	74.1

The genome-wide significance thresholds were F = 8.3 and F = 10.2 at the 5% and 1% level respectively, for traits analysed using a combined F_2 _and backcross analysis. The sensory traits were analysed using only F_2 _animals and for these the significance thresholds were F = 9.9 and F = 12.9 at the 5% and 1% genome-wide significance level, respectively.

Three *PRKAG3 *alleles, *RN*^- ^(R225Q), *rn*^+ ^(wild type) and rn* (V224I), were segregating in this family material. Allele frequencies in the F_2 _and backcross generations are presented in Table 2. As an internal positive control, we performed a QTL analysis of muscle glycogen content without including the *PRKAG3*-genotype as a fixed effect. This gave a significant QTL effect with an F-value for muscle glycogen content of over 100, which completely disappears when the *PRKAG3*-genotype is included as a fixed effect (data not shown). This confirms the high quality of the phenotypic data and an excellent matching of genotype and phenotype data.

**Table 2:** Allele frequencies at the *PRKAG3/RN*-locus in the back-cross (BC) and F_2 _generations of a Hampshire x Landrace cross.

*Allele*	*Definition*	*F*_2_	*BC*
*RN*^-^	V224 Q225	0.42	0.60
*rn*^+ ^(wild type)	V224 R225	0.36	0.16
rn*	I224 R225	0.22	0.24

**Figure 1:**
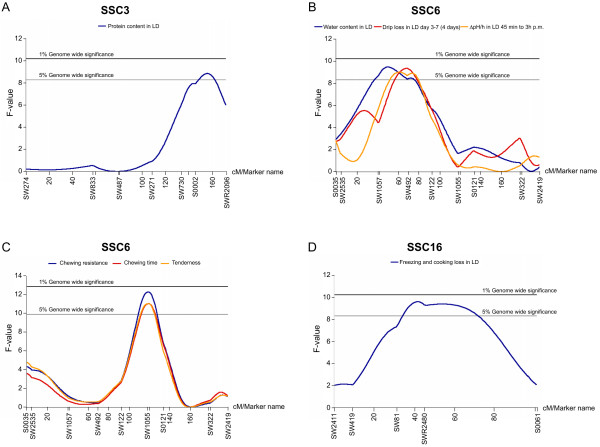
Test statistic curves for genome-wide significant QTLs. Horizontal lines indicate the 1% and 5% genome-wide significant thresholds applicable at the corresponding graph. Markers and distances in cM are indicated on the x-axis. A. QTL for protein content in LD on SSC3. B. QTLs on SSC6 for ΔpH/h in LD 45 min to 3 h p.m., drip loss in LD day 3–7 (4 days) and protein content in LD. C. QTLs for chewing resistance, chewing time and tenderness on SSC6. D. QTL for freezing and cooking loss in LD on SSC16. LD – *M. longissimus dorsi*, p.m.-*post mortem*

We performed genome scans for 39 meat quality traits (Table 3) and observed eight genome-wide significant QTL tests (Table 4; Figure 1). This is more than the expected number of Type I errors, given the fact that we have carried out genome scans for 39 traits and used the 5% significance level. All QTL tests that reached chromosome-wise significance are compiled in Table 5. We observed 46 suggestive QTLs which are only slightly more than the 39 expected Type I errors (~1 per genome scan/trait). Thus, a large proportion of the suggestive QTLs is expected to be false positives and further studies are needed to sort out which ones are true positives. The suggestive QTLs are therefore not further discussed here except those that were co-localized with QTLs showing genome-wide significance. No QTL showing genetic imprinting was detected in this study (data not shown).

**Table 3:** List of meat quality traits measured in a Hampshire x Landrace cross.

Trait		*n*	*Mean*	*SD*	*Reference*
pH:					
	LD 45 min p.m.	281	6.5	0.2	14
	LD 5 h p.m.	225	6.0	0.2	14
	LD 24 h p.m.	289	5.4	0.1	14,15
	LD 48 h p.m.	289	5.3	0.1	14
	SM 24 h p.m.	289	5.4	0.1	14
	BF 24 h p.m.	289	5.5	0.1	14
ΔpH/h:					
	45 min to 3 h p.m.	279	0.2	0.1	
	45 min to 5 h p.m.	216	0.1	0.0	
	3 h to 5 h p.m.	204	0.07	0.05	
Drip loss in LD, %:					
	day 3–4 p.m. (24 h)	289	3.7	1.0	14
	day 3–7 p.m. (4 days)	268	6.7	1.4	14
PSE spots, scale 0–3:					
	ham 24 h p.m.	289	0.4	0.6	14
	LD 24 h p.m.	289	0.1	0.3	14
Internal reflectance:					
	LD 24 h p.m.	289	26.3	5.9	14,15
	SM 24 h p.m.	289	36.6	6.4	14
	BF 24 h p.m.	289	41.3	7.1	14
Colour of LD:					
	NPPC, Japanese scale 1–6	288	2.9	0.5	
	L* (lightness)	289	56.3	1.8	15
	a* (redness)	289	6.6	1.2	15
	b* (yellowness)	289	15.1	0.9	15
Composition of LD:					
	Glycogen 24 h p.m., μmol/g DM	111	149	113	14
	Pigment content, mg hematine/kg	91	36.5	1.6	14
	Water content, %	175	76.3	0.8	14
	Intramuscular fat, %	175	0.8	0.3	14
	Protein content, %	175	21.6	1.1	14
Warner-Bratzler shear force in LD, N/cm^2^		289	68.7	21.8	14
Freezing and cooking loss in LD, %		289	30.2	2.8	14
Sensory evaluation by a panel (scale 1–100):					
	Appearance	53	51.9	7.3	22
	Chewing resistance	53	40.5	11.7	22
	Chewing time	53	56.3	9.9	22
	Tenderness	53	53.8	14.7	22
	Juiciness	53	62.6	5.5	22
	Flavour	53	57.4	3.9	22
	Acid	53	29.4	7.4	22
	Off-flavour	53	2.9	1.9	22
	Total impression	53	47.0	7.5	22
Male hormones in back fat:					
	Estrone, ng/g	138	1504	835	23
	Skatole, μg/g	139	0.1	0.1	23
	Androstenone, μg/g	139	1.1	1.6	23

LD – *M. longissimus dorsi*

SM – *M. semimembranosus*

BF – *M. biceps femoris*

p.m. – *post mortem*

DM – dry matter

*n *is the number of individuals with both phenotype and genotype recordings.

**Table 4:** QTL significant at the genome-wide level in a Hampshire x Landrace cross.

		*Position*	*95% CI*		*Additive*	*Dominance*		
*Chromosome/Trait*	*n*	*(cM)*	*(cM)*	*F-value*	*effect ± SE*	*effect ± SE*	*% Var*	*Model*
SSC3								
Protein content in LD	175	156	116–172	9.1*	0.4 ± 0.1	0.3 ± 0.2	10.1	HSRNT
								
SSC6								
Water content in LD	175	51	13–147	9.5*	0.4 ± 0.1	-0.3 ± 0.2	10.5	HSRNT
Drip loss in LD day 3–7 (4 days)	268	69	16–178	9.3*	0.6 ± 0.1	0.3 ± 0.2	6.9	HSRNT
ΔpH/h in LD 45 min to 3 h p.m.	279	61	0–89	9.0*	0.05 ± 0.01	0.01 ± 0.02	6.4	HSRNT
								
Chewing resistance	53	119	0–133	12.3*	9.9 ± 3.5	-25.1 ± 5.2	36.9	HRNT
Tenderness	53	119	0–131	11.0*	-11.3 ± 4.6	31.3 ± 6.8	34.4	HRNT
Chewing time	53	119	0–135	11.0*	8.8 ± 3.1	-20.5 ± 4.6	34.4	HRNT
								
SSC16								
Freezing and cooking loss in LD	289	41	25–78	9.6*	-0.0 ± 0.2	-1.2 ± 0.3	6.5	HSRNT

*5% genome-wide significance

LD – *M. longissimus dorsi*

p.m.-*post mortem*

CI – confidence interval

The additive effect was defined as the estimated phenotypic difference between animals homozygous for the Hampshire allele and the mean of the two homozygotes.

The dominance effect was calculated as the phenotypic deviation of the heterozygotes from the mean of the two homozygotes

Var – residual variance explained by the QTL.

*Model: *H = Herd, S = Sex, RN = *PRKAG3/RN*-genotype, T = Stunning procedure.

**Table 5:** QTL significant at the chromosome-wise level in a Hampshire x Landrace cross

*Chr*	*Trait*	*n*	*Position (cM)*	*F-value*	*Additive* *effect ± SE*	*Dominance* *effect ± SE*	*Model*
1	Estrone	138	123	5.6*	36 ± 157	-537 ± 199	HRNT
2	Acid	53	0	9.2**	-9.8 ± 2.4	13.8 ± 8.4	HRNT
	Water content in LD	175	0	5.5*	-0.3 ± 0.1	0.7 ± 0.4	HSRNT
	Warner-Bratzler shear force in LD	289	141	5.4*	5.5 ± 2.4	-4.5 ± 3.2	HSRNT
3	Estrone	138	0	6.8**	364 ± 260	-845 ± 385	HRNT
	Warner-Bratzler shear force in LD	289	2	6.2*	-9.3 ± 4.2	-22.3 ± 6.6	HSRNT
	Water content in LD	175	142	7.6*	-0.3 ± 0.1	-0.2 ± 0.1	HSRNT
	Colour a*	289	150	5.8*	0.2 ± 0.1	0.5 ± 0.2	HSRNT
	Log glycogen 24 h p.m.	111	166	7.1**	-0.01 ± 0.02	-0.13 ± 0.04	HSRNT
4	PSE spots in LD 24 h p.m.	289	115	4.9*	0.03 ± 0.05	0.2 ± 0.1	HSRNT
5	Colour NPPC	288	147	7.0**	0.2 ± 0.1	0.03 ± 0.09	HSRNT
6	Intramuscular fat in LD	175	54	7.0*	-0,2 ± 0.1	-0.1 ± 0.1	HSRNT
	Drip loss in LD day 3–4 p.m. (24 h)	289	58	7.3*	0.5 ± 0.1	0.5 ± 0.2	HSRNT
	Freezing and cooking loss in LD	289	70	8.3**	1.1 ± 0.3	0.7 ± 0.4	HSRNT
	Total impression	53	120	8.9*	-8.0 ± 2.5	13.7 ± 3.8	HRNT
	ΔpH/h 3 h to 5 h p.m.	204	147	5.6*	-0.03 ± 0.01	-0.03 ± 0.01	HSRNT
8	Colour NPPC	288	42	5.4*	-0.4 ± 0.1	-0.5 ± 0.2	HSRNT
9	Estrone	138	22	5.7*	394 ± 199	-411 ± 250	HRNT
	Chewing time	53	27	6.5*	-0.7 ± 2.9	-23.0 ± 6.5	HRNT
	Tenderness	53	29	6.7*	1.3 ± 4.2	33.3 ± 9.3	HRNT
	Total impression	53	32	8.0**	3.3 ± 2.0	15.6 ± 4.4	HRNT
10	ΔpH/h 45 min to 3 h p.m.	279	45	5.3*	-0.04 ± 0.01	-0.02 ± 0.02	HSRNT
	ΔpH/h 45 min to 5 h p.m.	216	41	4.6*	-0.02 ± 0.01	0.00 ± 0.01	HSRNT
	PSE spots ham 24 h p.m.	289	49	5.1*	-0.2 ± 0.1	-0.2 ± 0.1	HSRNT
	Internal reflectance SM 24 h p.m.	289	89	4.6*	-1.5 ± 1.2	-5.4 ± 1.8	HSRNT
	Colour a*	289	122	5.0*	0.1 ± 0.2	-0.9 ± 0.3	HSRNT
11	Estrone	138	5	4.4*	76 ± 193	620 ± 238	HRNT
	Log androstenone	139	95	5.4*	-0.1 ± 0.2	0.6 ± 0.3	HRNT
12	pH LD 5 h p.m.	225	50	8.1**	-0.1 ± 0.0	-0.2 ± 0.0	HSRNT
	ΔpH/h 45 min to 3 h p.m.	279	51	6.3*	0.03 ± 0.01	0.05 ± 0.01	HSRNT
	Internal reflectance BF 24 h p.m.	289	88	5.5*	3.3 ± 1.1	3.6 ± 1.4	HSRNT
13	Intramuscluar fat in LD	175	30	5.2*	0.06 ± 0.04	0.2 ± 0.1	HSRNT
	pH BF 24 h p.m.	289	47	7.2*	0.01 ± 0.01	0.07 ± 0.02	HSRNT
	Appearance	53	114	8.0*	7.8 ± 2.0	-1.8 ± 3.2	HRNT
	Acid	53	123	6.9*	3.8 ± 1.6	-7.6 ± 2.7	HRNT
14	Log androstenone	139	40	5.8**	0.3 ± 0.1	0.0 ± 0.1	HRNT
	Estrone	138	105	4.7*	-214 ± 187	406 ± 266	HRNT
15	Intramuscular fat in LD	175	0	6.1*	0.2 ± 0.1	0.4 ± 0.1	HSRNT
	pH LD 5 h p.m.	225	65	5.9*	0.1 ± 0.0	0.04 ± 0.04	HSRNT
	pH BF 24 h p.m.	289	75	6.6*	0.01 ± 0.02	0.07 ± 0.02	HSRNT
	PSE spots LD 24 h p.m.	289	76	6.7*	-0.2 ± 0.1	-0.04 ± 0.06	HSRNT
	PSE spots ham 24 h p.m.	289	91	5.6*	-0.0 ± 0.1	0.4 ± 0.1	HSRNT
16	Drip loss in LD day 3–7 p.m. (4 days)	268	31	6.9*	-0.07 ± 0.1	-0.6 ± 0.2	HSRNT
	Drip loss in LD day 3–4 p.m. (24 h)	289	31	5.2*	-0.1 ± 0.1	-0.4 ± 0.1	HSRNT
	Appearance	53	101	5.3*	-2.9 ± 2.5	-17.9 ± 5.8	HRNT
18	Log glycogen 2 h p.m.	111	12	8.5**	0.1 ± 0.0	-0.04 ± 0.04	HSRNT

**1% chromosome-wise significance

*5% chromosome-wise significance

LD – *M. longissimus dorsi*

SM – *M. semimembranosus*

BF – *M. biceps femoris*

p.m.-*post mortem*

The additive effect was defined as the estimated phenotypic difference between animals homozygous for the Hampshire allele and the mean of the two homozygotes.

The dominance effect was calculated as the phenotypic deviation of the heterozygotes from the mean of the two homozygotes.

*Model: *H = Herd, S = Sex, RN = *PRKAG3/RN*-genotype, T = Stunning procedure.

On SSC3, a QTL for protein content in *M. Longissimus dorsi *(LD) was detected with a peak at 156 cM. The QTL showed an additive effect and the Hampshire allele was associated with a higher protein content. In the same region of chromosome 3 we detected QTLs for glycogen content in LD, water content in LD and colour a* (redness), all of which reached chromosome-wise significance (Table 5). The Hampshire allele was associated with reduced glycogen and water content and higher degree of redness (colour a*).

QTLs affecting water content in LD, drip loss in LD during four days and pH decline in LD between 45 min and 3 hours *post mortem *were detected between positions 51 and 69 cM on SSC6 (Table 4; Figure 1). It is likely that these significant effects reflect the action of a single QTL. The QTL showed an additive effect and the Hampshire allele was associated with higher water content, drip loss and pH decline after slaughter. In the same interval, QTL tests with chromosome-wise significance for freezing and cooking loss, drip loss during 24 hours and intramuscular fat in LD were obtained (Table 5). We excluded the previously published porcine C1843T mutation in the ryanodine receptor gene *(RYR1) *[18] as a causative mutation for this QTL since it did not segregate in the pedigree discussed herein.

Another QTL on SSC6, with its peak at position 119 cM, was identified for three highly correlated traits, chewing resistance, tenderness and chewing time, scored by a trained sensory panel. The significance and estimated effects of this QTL must be interpreted with caution since only 53 animals were scored for these traits. This QTL showed overdominance, which means that the heterozygous class had the most extreme phenotypic value, and was associated with higher tenderness (Table 4). A suggestive QTL (1% chromosome-wise significance) for the total impression of the meat was found in the same region of chromosome 6 (Table 5).

We identified a QTL for freezing and cooking loss at 41 cM on SSC16 showing overdominance; the heterozygotes showed reduced freezing and cooking losses (Table 4). In the same region, suggestive QTLs for drip loss during 4 days and 24 hours were also identified (Table 5).

## Discussion

Meat quality is obviously of great importance in commercial pig breeding and it is a trait that is difficult and expensive to measure accurately on a large number of pigs in a progeny testing scheme. It is therefore of considerable interest to identify QTLs in experimental populations and exploit such loci by marker assisted selection (MAS) in breeding programs. Furthermore, the molecular characterization of genes controlling meat quality and meat content can provide new insights into muscle metabolism. This is illustrated by the identification of missense mutations in *RYR1 *[18] and *PRKAG3 *[13] that have major effects on lean meat content and meat quality, as well as by the point mutation in intron 3 of *IGF2 *[19] underlying a major QTL for muscle growth and lean meat content. Thus, further genetic studies of the QTLs reported here may lead to new basic knowledge as well as practical applications.

We identified two QTL regions on SSC6. The first QTL region, located at position 51–69 cM, affects water content in LD, drip loss in LD over four days and pH decline in LD between 45 min and 3 hours *post mortem*. Several other studies have also identified QTLs for meat quality traits in this region. QTLs for meat quality, stress resistance and carcass composition were mapped to SSC6 in crosses including the Piétrain breed. These QTLs are most likely explained by a mutation in *RYR1 *occurring at a high frequency in the Piétrain breed [10,20]. The pigs in our cross do not carry this mutation. Another study using non-carriers of the *RYR1 *mutation has also identified QTLs for meat quality traits in this region. Malek *et al*. identified a suggestive QTL for pH 24 hours *post mortem *in loin using a Berkshire x Yorkshire cross [8]. The location of this QTL is in the same region as our QTLs and they both showed an additive effect. However, Malek *et al*. did not detect QTLs for drip loss and cooking loss in this region even though these traits were included in their study.

The second QTL region on SSC6, with a peak at 119 cM, influenced chewing resistance, chewing time and tenderness. These traits are highly correlated and we assume that it is a single QTL that influences all three traits. A panel of individuals subjectively scored these traits and only 53 pigs were included. The small sample size reduces the power to detect QTLs for these traits and the results should be interpreted with caution. The QTL showed overdominance and was estimated to explain an astonishing ~35% of the residual variance, which could be an overestimation due to the few number of pigs analyzed. To put these results in perspective, we performed a QTL analysis for muscle glycogen content on chromosome 15 using the same 53 pigs to test if we could detect the segregation at the *RN *locus with this small number of pigs (*PRKAG3-genotype *was not included as a fixed effect and *PRKAG3 *was excluded as a marker in the linkage map). We obtained a statistically significant F-value of 11.0 at approximately the correct position (data not shown). This demonstrates that we can detect loci with major phenotypic effects using only 53 animals. Interestingly, Szyda *et al*. have reported a QTL for tenderness with an overlapping location to our QTL using a Norwegian commercial slaughter pig cross including Duroc, Norweigan Landrace and Yorkshire [21]. Further studies are required to find out whether our observation reflects a Type I error or a new major locus with an important effect on meat quality.

A QTL for protein content in LD was found at 156 cM on SSC3 and, to the best of our knowledge, no QTL with similar effect has previously been reported in this region. Similarly, we are not aware of any previously reported QTL with a strikingly similar effect to the one for freezing and cooking loss in LD that we mapped to position 41 cM on SSC16. Pierzchala *et al*. identified QTLs for conductivity, pH measurements and stress response on SSC16 in crosses between Meishan, Wild Boar and Piétrain but they did not detect a QTL for cooking loss even though this trait was scored [22]. Paszek *et al*. also detected a QTL for pH in muscle on SSC16 but did not see any QTL for muscle moisture in that region [5]. Our QTL had no significant effect on pH values.

## Conclusion

In this study 39 meat quality traits were analyzed and we identified eight QTLs at the genome-wide significance level. The QTLs were located in four regions, one on chromosome 3, two on chromosome 6 and one on chromosome 16. This was the first time the Hampshire breed was used in a QTL study of meat quality traits and it enabled us to detect two previously undetected QTLs on chromosome 3 and 16. We also identified two QTLs on chromosome 6 that coincide with QTLs detected in previous studies. One of the chromosome 6 QTLs is located in the same region as QTLs explained by the C1843T mutation in the ryanodine receptor (*RYR1*), however we have been able to exclude this as a causative mutation for our QTL. Several interesting QTL regions have been identified in this study and, although they require further investigation, they may be interesting for Marker Assisted Selection (MAS) in the future.

## Methods

### Animals and genotyping

The breeding company Quality Genetics established a three generation-cross between Finnish Landrace and Swedish Hampshire for commercial reasons. A combined intercross and backcross design was used. Eight Landrace boars (L) were crossed with 41 Hampshire sows (H) generating 52 animals in the F_1_-generation (LH). F_1_-animals were then intercrossed to produce 136 F_2_-animals. LH animals from the F_1_-generation were also reciprocally backcrossed to 42 purebred Hampshire pigs producing 112 (LH × H) and 72 (H × LH) offspring. Including the parental generation of the purebred Hampshire pigs the pedigree comprised a total of 527 animals. The offspring represented 86 full-sib families.

Husbandry and slaughtering as well as the phenotypic measurements have previously been described in detail [14-16,23]. The pigs were raised at three different breeding farms referred to as herd, but they were slaughtered at the same commercial slaughterhouse. During the experiment, the stunning procedure at the slaughterhouse changed, from individual stunning with CO_2 _to stunning in groups of five pigs. The traits analyzed in the current study are listed in Table 1.

A total of 120 microsatellite markers covering the autosomes were PCR amplified in 450 animals (excluding the 77 purebred Hampshire sows in the parental generation) and genotyped using either an ABI PRISM^® ^3100 Genetic Analyzer and ABI GeneMapper™ Genotyping Software in Copenhagen or a MegaBACE™ 1000 DNA Analysis System and Genetic Profiler (Amersham Biosciences) in Uppsala.

The three alleles, denoted *rn*^+ ^(wild type), *RN*^- ^(R225Q) and rn* (V224I), at the *PRKAG3/RN-*locus were scored according to a previously described method using pyrosequencing [13]. The single point mutation (C → T) in the pig *ryanodine receptor (ryr1) *gene changing an arginine to a cysteine at amino acid 615 [18] was genotyped with pyrosequencing using the following primers: forward primer with an M13-tag sequence CACGACGTTGTAAAACGACAGTGCCCTCACACCTTGAC, reverse primer CCAGGGAGCAAGTTCTCAGT, M13-biotinylated primer CACGACGTTGTAAAACGAC and sequencing primer AGTAATGAGATCTTGGTTGGAG. A 20 μl PCR reaction with 1× PCR Buffer II, 2.5 mM MgCl_2_, 0.3 mM dNTP, 0.03 μM forward primer, 0.3 μM of each reverse and M13-biotinylated primer, 0.75 U of AmpliTaq GOLD polymerase (Applied Biosystems) and 50–100 ng DNA was run using a standard touch-down PCR protocol. Starting with 95°C for 15 min, then 14 touch down cycles 95°C 30 s, 65–52°C 30 s, 72°C 30 s, followed by 30 cycles 95°C 30 s, 52°C 30 s, 72°C 30 s and ending with 72°C for 10 min. A standard pyrosequencing protocol was employed (Biotage).

### Statistical analysis

Linkage maps were built using the CRI-MAP program version 2.1 or 2.4 [24]. The sex average maps were used in the QTL analyses. Phenotypes were checked for normal distribution using the Ryan-Joiner normality test in MiniTab and transformed when necessary. The QTL analyses were performed using QTL express [25] and the combined F_2 _and backcross option as described in detail in our previous study [17]. The model including additive and dominance effects was compared with a model also including a parent-of-origin effect for all traits. For meat quality traits, except male hormones, the fixed effects herd, sex, stunning procedure and *PRKAG3/RN*-genotype were used; six different *PRKAG3/RN *genotypes were observed. For sex hormones, only herd, stunning procedure and *PRKAG3/RN*-genotype were included as fixed effects. The QTL analysis of sensory traits only included F_2 _progeny and these were therefore analyzed using the F_2 _design in QTL express. The model included herd, stunning procedure and *PRKAG3/RN*-genotype. Genome-wide significant thresholds were determined by permutation tests [26]. One thousand permutations were performed for all traits and an average calculated. Two different thresholds were permutated for traits analysed using the different options in QTL express. Chromosome-wise significant thresholds were also determined by permutation tests using thousand permutations. Confidence intervals (CI) were estimated for each genome-wide significant QTL using the bootstrap method [27] and 10,000 iterations were performed. For the genome-wide significant QTLs, the residual variance explained by the QTL was computed as ((Residual sums of squares reduced model – Residual sums of squares full model)/Residual sums of squares reduced model) ×100.

## Authors' contributions

EM compiled the Uppsala genotyping data, contributed to the QTL analyses, carried out the genotyping of the *RYR1 *mutation, summarized the data and drafted the manuscript. MHB carried out the DNA extractions and the genotyping in Uppsala. PKM contributed to the genotyping in Copenhagen and to the QTL analyses. CSB contributed to the genotyping in Copenhagen and to the QTL analyses. MS contributed to the genotyping in Copenhagen and to the QTL analyses. ICC contributed to retyping of some markers in Uppsala. IHV was responsible for the animal material and collection of samples for DNA extraction. ÅJ contributed to collection of the phenotypic data. KL contributed to the design of the study and to the collection of the phenotypic data. GvS contributed to collection of the phenotypic data. CBJ contributed to the genotyping in Copenhagen and to the QTL analyses. MF contributed to the design of the study and supervised this study in Copenhagen. LA designed the study and supervised the molecular analysis in Uppsala, edited and made final improvements to the manuscript. All authors read and approved the final manuscript.
